# *N*-(4-Meth­oxy­phen­yl)maleamic acid

**DOI:** 10.1107/S1600536810019999

**Published:** 2010-06-05

**Authors:** B. Thimme Gowda, Miroslav Tokarčík, K. Shakuntala, Jozef Kožíšek, Hartmut Fuess

**Affiliations:** a Department of Chemistry, Mangalore University, Mangalagangotri 574 199, Mangalore, India; b Faculty of Chemical and Food Technology, Slovak Technical University, Radlinského 9, SK-812 37 Bratislava, Slovak Republic; c Institute of Materials Science, Darmstadt University of Technology, Petersenstrasse 23, D-64287 Darmstadt, Germany

## Abstract

In the title compound, C_11_H_11_NO_4_, the asymmetric unit contains two unique mol­ecules, both of which are almost planar, with r.m.s. deviations of 0.047 and 0.059 Å. The dihedral angles between the benzene ring and the plane of maleamic acid unit are 3.43 (5) and 5.79 (3)° in the two mol­ecules. The mol­ecular structures are stabilized by a short intra­molecular O—H⋯O hydrogen bond within each maleamic acid unit. In the crystal, inter­molecular N—H⋯O hydrogen bonds link the mol­ecules into zigzag chains extending along [1
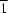
0]. Weak intermolecular C—H⋯O hydrogen bonds also exist.

## Related literature

For studies on the effect of ring- and side-chain substitutions on the crystal structures of amides, see: Gowda *et al.* (2009**a* ▶,*b* ▶,c* ▶); Prasad *et al.* (2002 ▶). For the modes of inter­linking carb­oxy­lic acids by hydrogen bonds, see: Jagannathan *et al.* (1994 ▶); Leiserowitz (1976 ▶).
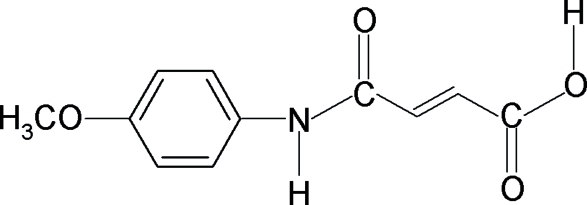


## Experimental

### 

#### Crystal data

C_11_H_11_NO_4_
*M* *_r_* = 221.21
Triclinic, 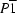

*a* = 7.34030 (17) Å
*b* = 11.8258 (4) Å
*c* = 12.1207 (4) Å
α = 89.103 (3)°
β = 88.358 (2)°
γ = 78.396 (2)°
*V* = 1030.15 (5) Å^3^
*Z* = 4
Mo *K*α radiation
μ = 0.11 mm^−1^
*T* = 295 K
0.54 × 0.25 × 0.22 mm

#### Data collection

Oxford Diffraction Gemini R CCD diffractometer
Absorption correction: analytical (*CrysAlis PRO*; Oxford Diffraction, 2009 ▶) *T*_min_ = 0.962, *T*_max_ = 0.980
15439 measured reflections
3711 independent reflections
2923 reflections with *I* > 2σ(*I*)
*R*_int_ = 0.020

#### Refinement

*R*[*F* ^2^ > 2σ(*F* ^2^)] = 0.035
*wR*(*F* ^2^) = 0.097
*S* = 1.02
3711 reflections
291 parameters
H-atom parameters constrained
Δρ_max_ = 0.14 e Å^−3^
Δρ_min_ = −0.20 e Å^−3^

### 

Data collection: *CrysAlis PRO* (Oxford Diffraction, 2009 ▶); cell refinement: *CrysAlis PRO*; data reduction: *CrysAlis PRO*; program(s) used to solve structure: *SHELXS97* (Sheldrick, 2008 ▶); program(s) used to refine structure: *SHELXL97* (Sheldrick, 2008 ▶); molecular graphics: *ORTEP-3* (Farrugia, 1997 ▶) and *DIAMOND* (Brandenburg, 2002 ▶); software used to prepare material for publication: *SHELXL97*, *PLATON* (Spek, 2009 ▶) and *WinGX* (Farrugia, 1999 ▶).

## Supplementary Material

Crystal structure: contains datablocks I, global. DOI: 10.1107/S1600536810019999/bq2215sup1.cif

Structure factors: contains datablocks I. DOI: 10.1107/S1600536810019999/bq2215Isup2.hkl

Additional supplementary materials:  crystallographic information; 3D view; checkCIF report

## Figures and Tables

**Table 1:** Hydrogen-bond geometry (Å, °)

*D*—H⋯*A*	*D*—H	H⋯*A*	*D*⋯*A*	*D*—H⋯*A*
O2—H2*A*⋯O1	0.92	1.55	2.4624 (13)	174
O6—H6*A*⋯O5	0.92	1.53	2.4466 (14)	177
N1—H1*N*⋯O7^i^	0.86	2.10	2.9305 (14)	162
N2—H2*N*⋯O3^ii^	0.86	2.10	2.9124 (14)	158
C2—H2⋯O6^i^	0.93	2.44	3.3592 (16)	171
C6—H6⋯O7^i^	0.93	2.52	3.2822 (17)	140
C22—H22⋯O2^ii^	0.93	2.51	3.3792 (16)	156
C26—H26⋯O3^ii^	0.93	2.57	3.2881 (16)	135
C11—H11*B*⋯O5^iii^	0.96	2.56	3.0688 (18)	113

Symmetry codes: (i) 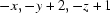; (ii) 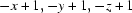; (iii) 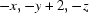.

**Fig. 1.**
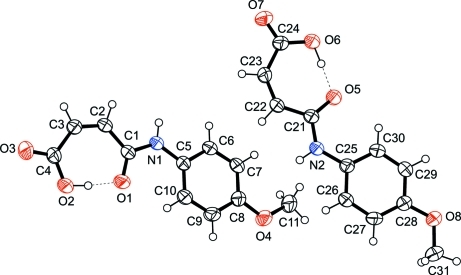
Molecular structure of (I) showing the atom labeling scheme. Displacement ellipsoids are drawn at the 50% probability level. Intramolecular O—H···O bonds are shown as dashed lines.

**Fig. 2.**
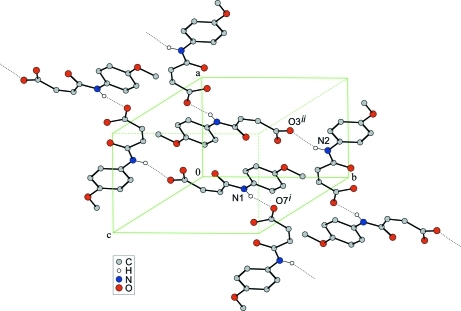
Part of the crystal structure of (I) showing zigzag chains of molecules linked by intermolecular N—H···O hydrogen bonds, represented by dashed lines. Symmetry codes (i): -x,-y + 2,-z + 1; (ii) -x + 1,-y + 1,-z + 1. H atoms not involved in intermolecular hydrogen bonding have been omitted.

## supplementary crystallographic information

### Comment

As a part of studying the effect of the ring and side chain substitutions on the crystal structures of biologically significant amides (Gowda *et al.*, 2009*a,b,c*; Prasad *et al.*, 2002), in the present work, the crystal structure of *N*-(4-methoxyphenyl)maleamic acid (I) has been determined (Fig. 1). The asymmetric unit of the structure contains two unique molecules.  Both the molecules are almost planar with r.m.s deviations of 0.047Å and 0.059Å for the two molecules (Fig. 1).

The conformations of the N—H and the C=O bonds in the amide segment are *anti* to each other. In the side chain, the amide C=O bond is *anti* to the adjacent C—H bond, while the carboxyl C=O bond is *syn* to the adjacent C—H bond. The observed rare *anti* conformation of the C=O and O—H bonds of the acid group is similar to that obsrved in *N*-(2,6-dimethylphenyl)-maleamic acid (Gowda *et al.*, 2009*a*), *N*-(3,4-dimethylphenyl)-maleamic acid (Gowda *et al.*, 2009*b*) and *N*-(2,4,6-trimethylphenyl)- maleamic acid (Gowda *et al.*, 2009*c*).

The dihedral angles between the phenyl ring and the plane of maleamic acid moiety are 3.43 (5)° and 5.79 (3)° in the first and second molecules, respectively. The molecular structure is stabilized by a short O–H···O intramolecular hydrogen bond (Table 1) within each maleamic acid moiety. The orientations of the methoxy groups toward the phenyl rings are given by the torsion angles, C7—C8—O4—C11 = -8.2 (2)° and C27—C28—O8—C31 = 0.7 (2)°. In the crystal structure (Fig. 2), the intermolecular N–H···O hydrogen bonds link the molecules into zigzag chains extending along the [1 -1 0] direction. Weak intermolecular C-H···O hydrogen bonds also exist in the structure.

The various modes of interlinking carboxylic acids by hydrogen bonds is described elsewhere (Leiserowitz, 1976). The packing of molecules involving dimeric hydrogen bonded association of each carboxyl group with a centrosymmetrically related neighbor has also been observed (Jagannathan *et al.*, 1994).

### Experimental

The solution of maleic anhydride (0.025 mol) in toluene (25 ml) was treated dropwise with the solution of 4-methoxyaniline (0.025 mol) also in toluene (20 ml) with constant stirring. The resulting mixture was stirred for about 30 min and set aside for an additional 30 min at room temperature for the completion of reaction. The mixture was then treated with dilute hydrochloric acid to remove the unreacted 4-methoxyaniline. The resultant solid *N*-(4-methoxyphenyl)maleamic acid was filtered under suction and washed thoroughly with water to remove the unreacted maleic anhydride and maleic acid. It was recrystallized to constant melting point from ethanol. The purity of the compound was checked by elemental analysis and characterized by its infrared spectra. Prism like yellowish green single crystals of the title compound used in X-ray diffraction studies were grown in an ethanol solution by slow evaporation at room temperature.

### Refinement

All H atoms were visible in difference maps and were further positioned with idealized geometry (C–H = 0.93 or 0.96 Å, N–H = 0.86 Å, O–H = 0.92 Å) and refined using a riding model. The *U*_iso_(H) values were set at 1.2*U*_eq_(C aromatic, N) and 1.5*U*_eq_(C methyl, O).

### Crystal data

**Table:**

C_11_H_11_NO_4_	*Z* = 4
*M**_r_* = 221.21	*F*(000) = 464
Triclinic, *P*1	*D*_x_ = 1.426 Mg m^−^^3^
Hall symbol: -P 1	Mo *K*α radiation, λ = 0.71073 Å
*a* = 7.34030 (17) Å	Cell parameters from 8581 reflections
*b* = 11.8258 (4) Å	θ = 1.8–29.5°
*c* = 12.1207 (4) Å	µ = 0.11 mm^−^^1^
α = 89.103 (3)°	*T* = 295 K
β = 88.358 (2)°	Prism, yellow–green
γ = 78.396 (2)°	0.54 × 0.25 × 0.22 mm
*V* = 1030.15 (5) Å^3^	

### Data collection

**Table:**

Oxford Diffraction Gemini R CCD diffractometer	3711 independent reflections
graphite	2923 reflections with *I* > 2σ(*I*)
Detector resolution: 10.434 pixels mm^-1^	*R*_int_ = 0.020
ω scans	θ_max_ = 25.2°, θ_min_ = 2.4°
Absorption correction: analytical (*CrysAlis PRO*; Oxford Diffraction, 2009)	*h* = −8→8
*T*_min_ = 0.962, *T*_max_ = 0.980	*k* = −14→14
15439 measured reflections	*l* = −14→14

### Refinement

**Table:**

Refinement on *F*^2^	Primary atom site location: structure-invariant direct methods
Least-squares matrix: full	Secondary atom site location: difference Fourier map
*R*[*F*^2^ > 2σ(*F*^2^)] = 0.035	Hydrogen site location: inferred from neighbouring sites
*wR*(*F*^2^) = 0.097	H-atom parameters constrained
*S* = 1.02	*w* = 1/[σ^2^(*F*_o_^2^) + (0.0619*P*)^2^ + 0.0566*P*] where *P* = (*F*_o_^2^ + 2*F*_c_^2^)/3
3711 reflections	(Δ/σ)_max_ < 0.001
291 parameters	Δρ_max_ = 0.14 e Å^−^^3^
0 restraints	Δρ_min_ = −0.20 e Å^−^^3^

### Special details

**Table:**

Geometry. All e.s.d.'s (except the e.s.d. in the dihedral angle between two l.s. planes) are estimated using the full covariance matrix. The cell e.s.d.'s are taken into account individually in the estimation of e.s.d.'s in distances, angles and torsion angles; correlations between e.s.d.'s in cell parameters are only used when they are defined by crystal symmetry. An approximate (isotropic) treatment of cell e.s.d.'s is used for estimating e.s.d.'s involving l.s. planes.
Refinement. Refinement of *F*^2^ against ALL reflections. The weighted *R*-factor *wR* and goodness of fit *S* are based on *F*^2^, conventional *R*-factors *R* are based on *F*, with *F* set to zero for negative *F*^2^. The threshold expression of *F*^2^ > σ(*F*^2^) is used only for calculating *R*-factors(gt) *etc*. and is not relevant to the choice of reflections for refinement. *R*-factors based on *F*^2^ are statistically about twice as large as those based on *F*, and *R*- factors based on ALL data will be even larger.

### Fractional atomic coordinates and isotropic or equivalent isotropic displacement parameters (Å^2^)

**Table:**

	*x*	*y*	*z*	*U*_iso_*/*U*_eq_	
C1	0.25076 (17)	0.51156 (11)	0.57066 (10)	0.0407 (3)	
C2	0.23780 (18)	0.54345 (11)	0.68793 (11)	0.0431 (3)	
H2	0.1732	0.6177	0.7035	0.052*	
C3	0.30661 (18)	0.47979 (12)	0.77505 (11)	0.0466 (3)	
H3	0.2826	0.5179	0.8421	0.056*	
C4	0.41425 (18)	0.35955 (12)	0.78463 (11)	0.0459 (3)	
C5	0.15402 (16)	0.58696 (10)	0.38503 (10)	0.0386 (3)	
C6	0.05175 (18)	0.68231 (11)	0.33150 (11)	0.0439 (3)	
H6	−0.0084	0.7452	0.3727	0.053*	
C7	0.03742 (18)	0.68568 (11)	0.21824 (11)	0.0457 (3)	
H7	−0.0314	0.7506	0.1838	0.055*	
C8	0.12554 (18)	0.59239 (11)	0.15582 (10)	0.0444 (3)	
C9	0.2264 (2)	0.49671 (12)	0.20906 (11)	0.0518 (4)	
H9	0.286	0.4337	0.1677	0.062*	
C10	0.24030 (19)	0.49303 (12)	0.32182 (11)	0.0487 (3)	
H10	0.3076	0.4275	0.3561	0.058*	
C11	0.0013 (2)	0.67727 (14)	−0.01187 (12)	0.0599 (4)	
H11A	0.0047	0.6609	−0.0893	0.09*	
H11B	0.0408	0.7488	−0.001	0.09*	
H11C	−0.1233	0.6832	0.0171	0.09*	
N1	0.16464 (14)	0.59247 (9)	0.50150 (8)	0.0406 (3)	
H1N	0.1085	0.656	0.5312	0.049*	
O1	0.33527 (15)	0.41551 (8)	0.53737 (8)	0.0625 (3)	
O2	0.45825 (14)	0.29623 (8)	0.69728 (8)	0.0593 (3)	
H2A	0.4196	0.3394	0.6353	0.089*	
O3	0.45985 (15)	0.32053 (9)	0.87533 (8)	0.0644 (3)	
O4	0.12243 (14)	0.58681 (9)	0.04387 (8)	0.0602 (3)	
C21	0.22420 (17)	0.98092 (11)	0.13550 (10)	0.0415 (3)	
C22	0.21905 (18)	0.94474 (11)	0.25224 (10)	0.0436 (3)	
H22	0.2751	0.8685	0.2671	0.052*	
C23	0.14486 (18)	1.00662 (12)	0.33956 (11)	0.0464 (3)	
H23	0.1513	0.9648	0.4055	0.056*	
C24	0.05379 (18)	1.13023 (12)	0.35040 (11)	0.0452 (3)	
C25	0.33278 (16)	0.91072 (11)	−0.05093 (10)	0.0390 (3)	
C26	0.40524 (19)	0.81103 (11)	−0.10879 (11)	0.0501 (3)	
H26	0.4333	0.7409	−0.071	0.06*	
C27	0.4366 (2)	0.81386 (12)	−0.22171 (11)	0.0512 (3)	
H27	0.4853	0.7461	−0.2593	0.061*	
C28	0.39523 (18)	0.91759 (11)	−0.27842 (10)	0.0448 (3)	
C29	0.3238 (2)	1.01727 (12)	−0.22083 (11)	0.0528 (4)	
H29	0.2962	1.0874	−0.2587	0.063*	
C30	0.2928 (2)	1.01469 (12)	−0.10878 (11)	0.0503 (3)	
H30	0.2448	1.0828	−0.0714	0.06*	
C31	0.4932 (2)	0.83253 (14)	−0.45312 (12)	0.0605 (4)	
H31A	0.5083	0.855	−0.5287	0.091*	
H31B	0.4089	0.7801	−0.4484	0.091*	
H31C	0.6116	0.7952	−0.4253	0.091*	
N2	0.30677 (14)	0.90022 (9)	0.06518 (8)	0.0425 (3)	
H2N	0.3498	0.8334	0.0934	0.051*	
O5	0.15438 (16)	1.08043 (9)	0.10395 (8)	0.0675 (3)	
O6	0.03939 (18)	1.19821 (9)	0.26512 (9)	0.0740 (4)	
H6A	0.0815	1.156	0.203	0.111*	
O7	−0.00459 (15)	1.16789 (9)	0.44012 (8)	0.0625 (3)	
O8	0.42056 (16)	0.93176 (9)	−0.38950 (8)	0.0622 (3)	

### Atomic displacement parameters (Å^2^)

**Table:**

	*U*^11^	*U*^22^	*U*^33^	*U*^12^	*U*^13^	*U*^23^
C1	0.0464 (7)	0.0308 (7)	0.0407 (7)	0.0016 (5)	0.0001 (5)	0.0018 (5)
C2	0.0502 (7)	0.0308 (7)	0.0424 (7)	0.0056 (5)	0.0015 (5)	0.0002 (6)
C3	0.0562 (8)	0.0393 (7)	0.0378 (7)	0.0058 (6)	−0.0007 (6)	−0.0002 (6)
C4	0.0510 (7)	0.0391 (7)	0.0426 (8)	0.0026 (6)	−0.0006 (6)	0.0064 (6)
C5	0.0423 (7)	0.0328 (7)	0.0381 (7)	−0.0017 (5)	0.0005 (5)	0.0024 (5)
C6	0.0551 (8)	0.0307 (7)	0.0415 (7)	0.0020 (6)	−0.0001 (6)	−0.0008 (6)
C7	0.0561 (8)	0.0318 (7)	0.0438 (8)	0.0038 (6)	−0.0042 (6)	0.0047 (6)
C8	0.0518 (8)	0.0410 (8)	0.0372 (7)	−0.0015 (6)	−0.0012 (6)	0.0007 (6)
C9	0.0604 (8)	0.0417 (8)	0.0436 (8)	0.0128 (6)	−0.0003 (6)	−0.0046 (6)
C10	0.0566 (8)	0.0375 (7)	0.0438 (8)	0.0101 (6)	−0.0027 (6)	0.0028 (6)
C11	0.0771 (10)	0.0557 (9)	0.0396 (8)	0.0045 (7)	−0.0068 (7)	0.0071 (7)
N1	0.0487 (6)	0.0303 (5)	0.0375 (6)	0.0045 (4)	0.0011 (4)	0.0013 (5)
O1	0.0925 (8)	0.0384 (6)	0.0427 (6)	0.0204 (5)	−0.0047 (5)	−0.0015 (4)
O2	0.0821 (7)	0.0365 (5)	0.0472 (6)	0.0170 (5)	−0.0037 (5)	0.0029 (5)
O3	0.0822 (7)	0.0526 (6)	0.0468 (6)	0.0141 (5)	−0.0062 (5)	0.0118 (5)
O4	0.0797 (7)	0.0535 (6)	0.0361 (5)	0.0137 (5)	−0.0050 (5)	−0.0008 (5)
C21	0.0456 (7)	0.0337 (7)	0.0403 (7)	0.0037 (5)	0.0009 (5)	−0.0005 (6)
C22	0.0523 (7)	0.0307 (7)	0.0420 (7)	0.0050 (5)	0.0017 (6)	0.0036 (6)
C23	0.0565 (8)	0.0392 (7)	0.0381 (7)	0.0026 (6)	0.0034 (6)	0.0050 (6)
C24	0.0504 (7)	0.0392 (7)	0.0413 (8)	0.0020 (6)	0.0032 (6)	−0.0032 (6)
C25	0.0416 (7)	0.0351 (7)	0.0369 (7)	0.0002 (5)	−0.0007 (5)	−0.0011 (5)
C26	0.0687 (9)	0.0329 (7)	0.0424 (8)	0.0043 (6)	0.0006 (6)	0.0018 (6)
C27	0.0699 (9)	0.0348 (7)	0.0429 (8)	0.0036 (6)	0.0022 (6)	−0.0081 (6)
C28	0.0552 (8)	0.0406 (8)	0.0367 (7)	−0.0048 (6)	−0.0002 (6)	−0.0021 (6)
C29	0.0748 (9)	0.0344 (7)	0.0436 (8)	0.0015 (6)	0.0044 (7)	0.0038 (6)
C30	0.0675 (9)	0.0332 (7)	0.0443 (8)	0.0030 (6)	0.0069 (6)	−0.0036 (6)
C31	0.0817 (10)	0.0549 (9)	0.0413 (8)	−0.0047 (8)	0.0054 (7)	−0.0117 (7)
N2	0.0533 (6)	0.0313 (6)	0.0375 (6)	0.0041 (5)	0.0010 (5)	0.0014 (5)
O5	0.1026 (8)	0.0409 (6)	0.0420 (6)	0.0240 (5)	0.0096 (5)	0.0054 (5)
O6	0.1190 (9)	0.0380 (6)	0.0475 (6)	0.0228 (6)	0.0169 (6)	0.0035 (5)
O7	0.0846 (7)	0.0484 (6)	0.0447 (6)	0.0092 (5)	0.0093 (5)	−0.0084 (5)
O8	0.0996 (8)	0.0456 (6)	0.0354 (5)	−0.0009 (5)	0.0053 (5)	−0.0028 (4)

### Geometric parameters (Å, °)

**Table:**

C1—O1	1.2468 (15)	C21—O5	1.2444 (15)
C1—N1	1.3341 (16)	C21—N2	1.3295 (16)
C1—C2	1.4719 (18)	C21—C22	1.4735 (18)
C2—C3	1.3377 (18)	C22—C23	1.3353 (18)
C2—H2	0.93	C22—H22	0.93
C3—C4	1.4858 (18)	C23—C24	1.4862 (19)
C3—H3	0.93	C23—H23	0.93
C4—O3	1.2151 (16)	C24—O7	1.2151 (16)
C4—O2	1.3015 (16)	C24—O6	1.2930 (17)
C5—C6	1.3858 (17)	C25—C26	1.3845 (18)
C5—C10	1.3921 (18)	C25—C30	1.3890 (18)
C5—N1	1.4193 (16)	C25—N2	1.4217 (16)
C6—C7	1.3792 (18)	C26—C27	1.3824 (18)
C6—H6	0.93	C26—H26	0.93
C7—C8	1.3853 (19)	C27—C28	1.3803 (19)
C7—H7	0.93	C27—H27	0.93
C8—O4	1.3606 (16)	C28—O8	1.3662 (16)
C8—C9	1.3829 (19)	C28—C29	1.3812 (19)
C9—C10	1.3728 (19)	C29—C30	1.3715 (19)
C9—H9	0.93	C29—H29	0.93
C10—H10	0.93	C30—H30	0.93
C11—O4	1.4213 (17)	C31—O8	1.4174 (17)
C11—H11A	0.96	C31—H31A	0.96
C11—H11B	0.96	C31—H31B	0.96
C11—H11C	0.96	C31—H31C	0.96
N1—H1N	0.86	N2—H2N	0.86
O2—H2A	0.92	O6—H6A	0.92
			
O1—C1—N1	121.77 (12)	O5—C21—N2	121.73 (11)
O1—C1—C2	122.78 (12)	O5—C21—C22	122.37 (12)
N1—C1—C2	115.45 (11)	N2—C21—C22	115.89 (11)
C3—C2—C1	128.61 (12)	C23—C22—C21	128.78 (12)
C3—C2—H2	115.7	C23—C22—H22	115.6
C1—C2—H2	115.7	C21—C22—H22	115.6
C2—C3—C4	131.96 (13)	C22—C23—C24	131.60 (13)
C2—C3—H3	114	C22—C23—H23	114.2
C4—C3—H3	114	C24—C23—H23	114.2
O3—C4—O2	120.22 (12)	O7—C24—O6	119.93 (12)
O3—C4—C3	119.17 (13)	O7—C24—C23	119.81 (13)
O2—C4—C3	120.61 (11)	O6—C24—C23	120.25 (12)
C6—C5—C10	118.38 (12)	C26—C25—C30	118.52 (12)
C6—C5—N1	117.26 (11)	C26—C25—N2	117.52 (11)
C10—C5—N1	124.36 (11)	C30—C25—N2	123.95 (11)
C7—C6—C5	121.26 (12)	C27—C26—C25	121.19 (12)
C7—C6—H6	119.4	C27—C26—H26	119.4
C5—C6—H6	119.4	C25—C26—H26	119.4
C6—C7—C8	120.00 (12)	C28—C27—C26	119.77 (12)
C6—C7—H7	120	C28—C27—H27	120.1
C8—C7—H7	120	C26—C27—H27	120.1
O4—C8—C9	116.12 (12)	O8—C28—C27	125.33 (12)
O4—C8—C7	124.99 (12)	O8—C28—C29	115.50 (12)
C9—C8—C7	118.89 (12)	C27—C28—C29	119.17 (12)
C10—C9—C8	121.19 (13)	C30—C29—C28	121.17 (13)
C10—C9—H9	119.4	C30—C29—H29	119.4
C8—C9—H9	119.4	C28—C29—H29	119.4
C9—C10—C5	120.27 (12)	C29—C30—C25	120.18 (12)
C9—C10—H10	119.9	C29—C30—H30	119.9
C5—C10—H10	119.9	C25—C30—H30	119.9
O4—C11—H11A	109.5	O8—C31—H31A	109.5
O4—C11—H11B	109.5	O8—C31—H31B	109.5
H11A—C11—H11B	109.5	H31A—C31—H31B	109.5
O4—C11—H11C	109.5	O8—C31—H31C	109.5
H11A—C11—H11C	109.5	H31A—C31—H31C	109.5
H11B—C11—H11C	109.5	H31B—C31—H31C	109.5
C1—N1—C5	128.19 (11)	C21—N2—C25	128.07 (11)
C1—N1—H1N	115.9	C21—N2—H2N	116
C5—N1—H1N	115.9	C25—N2—H2N	116
C4—O2—H2A	109.5	C24—O6—H6A	109.5
C8—O4—C11	117.28 (11)	C28—O8—C31	118.13 (11)
			
O1—C1—C2—C3	−1.0 (2)	O5—C21—C22—C23	−0.1 (2)
N1—C1—C2—C3	178.62 (13)	N2—C21—C22—C23	−179.46 (13)
C1—C2—C3—C4	−0.6 (2)	C21—C22—C23—C24	−3.5 (2)
C2—C3—C4—O3	−179.27 (15)	C22—C23—C24—O7	179.83 (14)
C2—C3—C4—O2	0.2 (2)	C22—C23—C24—O6	−1.4 (2)
C10—C5—C6—C7	−1.04 (19)	C30—C25—C26—C27	−0.4 (2)
N1—C5—C6—C7	178.47 (11)	N2—C25—C26—C27	−179.36 (12)
C5—C6—C7—C8	0.3 (2)	C25—C26—C27—C28	0.0 (2)
C6—C7—C8—O4	−179.29 (12)	C26—C27—C28—O8	179.68 (13)
C6—C7—C8—C9	0.3 (2)	C26—C27—C28—C29	0.3 (2)
O4—C8—C9—C10	179.56 (12)	O8—C28—C29—C30	−179.67 (12)
C7—C8—C9—C10	0.0 (2)	C27—C28—C29—C30	−0.2 (2)
C8—C9—C10—C5	−0.8 (2)	C28—C29—C30—C25	−0.1 (2)
C6—C5—C10—C9	1.27 (19)	C26—C25—C30—C29	0.4 (2)
N1—C5—C10—C9	−178.21 (12)	N2—C25—C30—C29	179.36 (12)
O1—C1—N1—C5	−0.7 (2)	O5—C21—N2—C25	1.0 (2)
C2—C1—N1—C5	179.61 (10)	C22—C21—N2—C25	−179.59 (11)
C6—C5—N1—C1	178.55 (12)	C26—C25—N2—C21	−173.12 (12)
C10—C5—N1—C1	−2.0 (2)	C30—C25—N2—C21	8.0 (2)
C9—C8—O4—C11	172.28 (13)	C27—C28—O8—C31	0.7 (2)
C7—C8—O4—C11	−8.2 (2)	C29—C28—O8—C31	−179.88 (12)

### Hydrogen-bond geometry (Å, °)

**Table:**

*D*—H···*A*	*D*—H	H···*A*	*D*···*A*	*D*—H···*A*
O2—H2A···O1	0.92	1.55	2.4624 (13)	174
O6—H6A···O5	0.92	1.53	2.4466 (14)	177
N1—H1N···O7^i^	0.86	2.10	2.9305 (14)	162
N2—H2N···O3^ii^	0.86	2.10	2.9124 (14)	158
C2—H2···O6^i^	0.93	2.44	3.3592 (16)	171
C6—H6···O7^i^	0.93	2.52	3.2822 (17)	140
C22—H22···O2^ii^	0.93	2.51	3.3792 (16)	156
C26—H26···O3^ii^	0.93	2.57	3.2881 (16)	135
C11—H11B···O5^iii^	0.96	2.56	3.0688 (18)	113

Symmetry codes: (i) −*x*, −*y*+2, −*z*+1; (ii) −*x*+1, −*y*+1, −*z*+1; (iii) −*x*, −*y*+2, −*z*.
